# Preliminary investigation of intraperitoneal raltitrexed in patients with gastric cancer

**DOI:** 10.1186/1477-7819-12-403

**Published:** 2014-12-30

**Authors:** Ping Zhao, Zhi Ding, Lingchao Tang, Xiang Zhou

**Affiliations:** Division of Gastrointestinal Surgery, Department of abdominal Surgery, Sichuan Cancer Hospital, No.55 of Renmin South Rd., Chengdu, 610041 Sichuan Province China

**Keywords:** Gastric cancer, Raltitrexed, Intraperitoneal therapy, Peritoneal perfusion, Patient safety

## Abstract

**Background:**

Peritoneal implantation metastasis of gastric cancer is the major reason for cancer recurrence after radical operations. As a new chemotherapeutic agent, raltitrexed has been widely used in intravenous chemotherapy for many kinds of cancers. However, no study has reported the efficacy and safety of raltitrexed in intraperitoneal chemotherapy. This study aimed to explore the safety of intraperitoneal chemotherapy with raltitrexed during gastric cancer operation compared to normal saline (NS) rinsing of the abdominal cavity.

**Methods:**

In this prospective study, 91 gastric cancer patients undergoing surgery and reconstruction were consecutively enrolled and randomly assigned into two groups. Raltitrexed in NS (500 ml) was injected into the abdominopelvic cavity for the patients in the RT group (n = 48), while for the patients in the group NS (n = 43), only NS (500 ml) was injected. The postoperative complications, gas passage time, and adverse effects, according to **NCI-CTCAE v3.0**, were compared between the two groups.

**Results:**

There were no significant differences in age, sex, cancer pathological type, clinical stage or operation method between the two groups (all *P* >0.05). No significant difference was observed in adverse effects and postoperative complications between the two groups (all *P* >0.05). No significant change was found in the levels of red blood cells, white blood cells, platelets, lactate dehydrogenase, blood urea nitrogen, and alanine aminotransferase before and after the operation for both groups (all *P* >0.05). All adverse events were mild or moderate by **NCI-CTCAE v3.0** (National Cancer Institute common terminology criteria for adverse events) grade.

**Conclusions:**

The findings of the present study demonstrate that intraperitoneal chemotherapy with raltitrexed after gastric cancer operation is safe and could be used for patients.

## Background

Gastric cancer is one of the most common malignancies in the world, and radical operations have been accepted as the best choice for treating gastric cancer [1]. The development of surgical techniques and equipment in the past two decades has also improved the treatment of gastric cancer, which has in turn improved the survival rate and quality of life of the patients. Gastric cancer surgery these days is performed based on cytological evidence rather than the presence of lumps alone. Further surgery ensures that there is no iatrogenic planting of the tumor and provides radical treatment in cytological terms. Based on cytological features, gastric cancer operation outcome will include either advanced gastric cancer with residual cancer (including residual cancer seen by the naked eye and small cancerous lesions or single cancer cells that cannot be observed by the naked eye) or early gastric cancer without residual cancer, regardless of the pre- or postoperative stages of the cancers [2–5]. However, the examination methods employed in the present study restricted us from discriminating micrometastasis from no metastasis.

Studies have reported that more than 70% of Chinese patients are diagnosed with stage III or IV gastric cancer at diagnosis and cannot be treated with radical surgery. Therefore, a high proportion of patients have to be treated with palliative surgery. In addition, radical operation cannot completely cure all patients. Surgical treatment can only remove the tumors and adjacent lymph nodes with metastatic cancer that can be visually identified; however, cancer cells or cell clones that are spread into other organs by invasion, hematogenous dissemination, or through the lymphatic system cannot be treated with operations. Therefore, even radical operation cannot prevent the recurrence or distant metastasis of the cancers. The limited cancer cells that have entered the lymph nodes could either proliferate to a large metastatic tumor or be eliminated by the immune system; in addition, several cells could also enter the G_0_ phase and proliferate into a large metastatic tumor under appropriate conditions [3–6].

Micrometastasis does not necessarily mean a poor outcome but the free cancer cells in the abdominal cavity of the patients are ‘time bombs’ that have the capacity to proliferate and induce the recurrence and peritoneal implantation metastasis [2]. Thus it is very important to prevent the micrometastasis. One of the first steps in this direction is to increase the detection rate. However, the sensitivity and specificity of different methods vary substantially with the peritoneal metastases. Metastases that can be observed with the naked eye are easy to detect, while micrometastasis means the cancer cells are generally in the blood circulation, lymphatic vessels, bone marrows, or other organs, and the sizes of cell clones are <2 mm or have even not been formed, and cannot be effectively detected by conventional examination including imaging or pathological examinations [2]. Furthermore, no obvious clinical symptoms can suggest micrometastasis. All these facts greatly limit our knowledge about metastatic gastric cancers. Fortunately, with the advance of biochemical technologies and new tumor biomarkers, the methods of detecting micrometastasis have also greatly improved. These have also brought forth more kinds of detection methods, for example, the traditionally used serial sectioning method was replaced by immunohistochemistry in the late 1980s, and application of polymerase chain reaction (PCR) has further increased the detection rates [3, 6–10].

Free cancer cells in the patient’s abdominal cavity are mainly from cancer tissues, and when cancer cells invade the gastric serosa they can also drop into the abdominal cavity due to the reduced adhesive force among the cancer cells; in addition, free cancer cells could also come from micrometastasis in the lymphatic system due to the resection of lymphatic vessels during surgery. These cancer cells that are free in the peritoneum could induce the recurrence or metastasis of gastric tumors [6, 11]. In the past two decades, free cancer cells in the peritoneal lavage of gastric cancer patients have been used as an important specimen by Japanese researchers. In a study performed by Kostic *et al*. [4], peritoneal lavage of 100 patients with gastric cancer was used for the diagnosis of the disease, and the detection rate was 24%, which is consistent with the results reported by Chuwa *et al*. (35.4%) [5] and Kodera *et al*. (24 to 39%, mainly 14 to 21%) [12]. However, this method also involves relatively high rates of false negatives [13]. In recent years, the development of RT-PCR and the advancement of different tumor biomarkers, especially keratin, have greatly improved the sensitivity and detection rate of the detection methods [14, 15]. Intraperitoneal administration of chemotherapeutic agents to eliminate the cancer cells can prevent tumor recurrence [8–11]. Intraperitoneal chemotherapy allows the direct reaction between the drugs and the surface of the peritoneum and the organs in the abdominal cavity, thus the cancer cells that have dropped from cancer tissues or other small cancer cell clones can be effectively killed, which in turn can prevent the local recurrence of gastric cancer. Many studies have investigated the methods and drugs used in preventing peritoneum metastasis. Several agents including cisplatin, 5-FU, hydroxycamptothecin, and slow-release Sinofuan have been used for intraperitoneal chemotherapy [8–11]. But more recently, perioperative intravenous chemotherapy has more often been the preferred treatment [16].

Occasional studies have included raltitrexed, a chemotherapeutic drug with a long half-life, in the treatment combination used for intravenous chemotherapy of gastric cancer [17, 18]. In those cases, the intravenous dosing regimens range from 1 mg/m^2^ to 3 mg/ml^2^ and were well tolerated, although one study suggested that there was no substantial antitumor activity using raltitrexed for gastric cancer intravenously [18]. Pharmacokinetic analysis of raltitrexed in cancer therapy has shown that dose limiting toxicity occurred at 4.8 and 7.5 mg/m^2^/day and that the maximum tolerated dose was 12 to 16 mg/m^2^/day [19]. Animal models of intraperitoneal administration of raltitrexed suggest that it is nontoxic at 1 and 2 mg doses in pigs and up to 8 mg/m^2^ in rats [20, 21]. Based on this information and our experience, raltitrexed has been used in intraperitoneal chemotherapy, at a dose of 4 mg, for gastric cancer patients after radical operation between January and July 2013 in our hospital. This study investigated the safety of raltitrexed for use in a preliminary report on this chemotherapy method.

## Methods

### Patients

Ninety-one patients with gastric cancer who had been treated in the department of Gastric Surgery, Cancer Hospital of Sichuan Province, between January 2013 and July 2013 were included in this study. Sixty-two of these patients were males, and 29 were females. The ages of the patients ranged from 37 to 72 years.

The inclusion criteria were patients with 1) invasion of the adjacent tissues or extraserosal areas were identified during the gastric cancer operation; 2) patients with obvious or possible residual cancer cells; and 3) patients who were treated with a palliative or radical operation.

The exclusion criteria were patients with diabetes, more than 75 years of age, who had not received chemotherapy before the operation, or who were unsuitable for chemotherapy.

This study was approved by the ethics committee of the Cancer Hospital of Sichuan Province. All included patients provided informed consent.

### Study design

The patients were randomly assigned into two groups by a computer-generated random number table, namely the raltitrexed (RT) group (48 patients) and the normal saline (NS) group (43 patients).

Conventional open operation was performed for all the patients. No-tumor procedures (including applying incise drape to the incisions, exploring the no-tumor regions prior to exploring the tumor regions, minimizing touching of the tumor, avoiding applying pressure to the tumor and ligating the vessels around the tumors first) were performed strictly to avoid iatrogenic planting and spread. After the gastric cancer had been removed and reconstruction had been performed, 2,000 to 2,500 ml of distilled water was used to rinse the abdominal cavity before it was closed. Then 4 mg of raltitrexed in 500 ml of normal saline (NS) was injected into the abdominal cavity for the patients in the RT group, while no chemotherapeutic agent but only 500 ml of NS was injected for the patients in the NS group. The injection solutions were prepared prior to the surgery and were administered according to the randomization by the surgeon who was blinded to the study groupings. Raltitrexed dosage was administered according to the manufacturer’s instructions that 3 mg/square meter be supplied as 500 ml 0.9% NS injection. The drainage tube was clamped for 2 hours after the operation to prevent the chemotherapy drugs from flowing from the peritoneal cavity.

### Safety evaluation

The effects of the treatments on the recovery, abdominal cavity and functions of other organs were evaluated according to the common toxicity criteria issued by the National Cancer Institute [2]. Complications including fever for more than 3 days, pulmonary infection, and anastomotic leakage were recorded. In addition, other parameters including gas passage time, drug allergy, abdominal drainage volume, peritoneal irritation signs (including abdominal pain and pressing pain), gastrointestinal toxic reaction (including vomit, diarrhea, and hemorrhage), hematologic toxic reaction (including white blood cell count, red blood cell count, and platelet count before and 7 days after the operation), renal toxicity (including elevation of blood urea nitrogen (BUN) and creatinine), and hepatotoxicity (including elevation of enzymes like alanine aminotransferase) were also measured. Adverse events were reported according to the National Cancer Institute common terminology criteria for adverse events (**NCI-CTCAE v3.0**; http://ctep.cancer.gov).

### Statistical analysis

SPSS 16.0 software (SPSS Inc., Chicago, Illinois, USA) was used for the statistical analysis. Quantitative data were described by means and standard deviations, and analyzed by Student’s unpaired *t*-test between groups, whereas qualitative data were described by proportions and analyzed by Chi-square test. *P* <0.05 was considered statistically significant.

## Results

### Clinical characteristics

The clinical characteristics of the patients in the RT and NS groups are listed in Table 1. No significant difference was found in age, sex, pathological type, clinical stages, and operation method between the two groups (*P* >0.05). Thus, the background data for all patients was relatively similar.

**Table 1:** **Baseline clinical characteristics of the 91 patients**

Parameter	Raltitrexed (RT) group n = 48	Normal saline (NS) group n = 43	***P*** value
Age (years)	56.4 ± 12.8	54.3 ± 11.2	>0.05
Sex (Males : Females)	41 : 7	37 : 6	>0.05
**Pathological type**			>0.05
Poorly differentiated adenocarcinoma	14	11	
Moderately differentiated	6	5	
Well-differentiated	7	7	
Mucoid carcinoma	11	10	
Signet-ring cell	8	9	
Papillary carcinoma	2	1	
**Clinical stages**			>0.05
Ib	1	0	
II	9	10	
IIIa	16	12	
IIIb	14	15	
IV	8	6	
**Operation methods**			>0.05
Distal gastrectomy	17	15	
Proximal gastrectomy	6	7	
Total gastrectomy	25	21	

### Incidence of postoperative complications

Incidences of postoperative complications in the RT and NS groups are listed in Table 2. No significant difference was found in postoperative fever, pulmonary infection, incision infection, gas passage time, abdominal drainage volume, and peritoneal irritation signs between the two groups (*P* >0.05). No anastomotic leakage was found in either of the two groups.

**Table 2:** **Postoperative complications in the patients in the two groups**

Parameter	Raltitrexed group (RT) n = 48	Normal saline group (NS) n = 43	P value
Pulmonary infection	2	2	>0.05
Incision infection	2	3	>0.05
Anastomotic leakage	0	0	
Gas passage time (hours)	3.43 ± 0.92	3.54 ± 0.86	>0.05
Abdominal drainage volume (ml)	230 ± 60	180 ± 90	>0.05
Peritoneal irritation signs	2	1	>0.05

### Toxic effects in the two groups

Toxic effects in the RT and NS groups are listed in Table 3. When assessing hematologic toxicity, it was found that there were no significant differences in parameters like red blood cell (RBC), white blood cell (WBC), and platelet counts between the two groups, either before or after the operation (*P* >0.05). One patient in group A had the lowest granulocyte count of 1.2 × 10^9^/L and platelet count of 3 × 10^9^/L; another patient in the group B had a granulocyte count of 2.2 × 10^9^/L and platelet count of 6 × 10^9^/L. Drugs were used to stimulate the proliferation of white blood cells for these two patients, and the granulocyte count and hemoglobin level recovered to normal levels at 14-day postoperation. No significant difference in BUN, alanine aminotransferase (ALT), lactate dehydrogenase (LDH), and gastrointestinal toxic reactions between the two groups, either before or after chemotherapy (*P* >0.05). No congestive heart failure, interstitial pneumonia (proven by X-ray), central coma, or peripheral numbness was found in either group. However, one case of drug rash was found in each of the two groups.

**Table 3:** **Toxic and adverse effects in the patients in the two study groups**

Parameter	Raltitrexed group (RT) n = 48	Normal saline group (NS) n = 43	***P*** value
Hematologic toxicity			
**RBC (10** ^**12**^ **/L)**			
Preoperative	3.68 ± 0.22	3.76 ± 0.14	>0.05
Postoperative	3.49 ± 0.21	3.56 ± 0.24	>0.05
**WBC (10** ^**9**^ **/L)**			
Preoperative	4.73 ± 0.46	4.45 ± 0.54	>0.05
Postoperative	6.29 ± 0.63	6.70 ± 0.52	>0.05
**Platelet count (10** ^**9**^ **/L)**			
Preoperative	183.09 ± 27.19	178.22 ± 28.24	>0.05
Postoperative	196.15 ± 25.28	192.36 ± 23.75	>0.05
**Renal toxicity BUN (mmol/L)**			
Preoperative	4.38 ± 0.72	4.46 ± 0.75	>0.05
Postoperative	4.87 ± 1.04	4.68 ± 0.84	>0.05
**Renal toxicity Creatinine (μmol/L)**			
Preoperative	76.24 ± 7.67	73.46 ± 6.95	>0.05
Postoperative	78.37 ± 7.44	74.68 ± 7.14	>0.05
**Hepatotoxicity (U/L)**			
ALT			
Preoperative	21.36 ± 2.78	19.92 ± 2.84	>0.05
Postoperative	24.35 ± 3.02	22.01 ± 2.65	>0.05
LDH (mmol/L)			
Preoperative	186.46 ± 27.19	192.34 ± 32.45	>0.05
Postoperative	191.24 ± 26.76	190.56 ± 30.12	>0.05

Note: One case was found with low granulocyte count in each of the two groups, and another case in group A was found with low hemoglobin level.

ALT, alanine aminotransferase; BUN, blood urea nitrogen; LDH, lactate dehydrogenase; RBC, red blood cell; WBC, white blood cell.

When toxic and adverse events were classified according to the **NCI-CTCAE v3.0**, there were no significant differences between the groups (Table 4). The most common were eight cases of abdominal ache in the RT group and nine cases in the NS group; six cases of hepatotoxicity in the RT group and five cases in the NS group; and five cases of infection in the RT group and six cases in the NS group. All of the reported events were grade I or II and therefore were mild or moderate.

**Table 4:** **Toxic and adverse effects in patients in the two study groups (According to NCI-CTCAE v3.0)**

Parameter	Raltitrexed group (RT) n = 48					Normal saline group (NS) n = 43				
	I	II	III	IV	V	I	II	III	IV	V
**WBC**	3	2	1	0	0	3	2	0	0	0
**Platelet count**	2	2	0	0	0	3	2	0	0	0
**Bleeding**	0	0	0	0	0	0	0	0	0	0
**Renal toxicity**	3	0	0	0	0	3	0	0	0	0
**Hepatotoxicity**	5	1	0	0	0	4	1	0	0	0
**Fever (postoperation >7 days)**	0	0	0	0	0	0	0	0	0	0
**Infection**	5	0	0	0	0	6	0	0	0	0
**Diarrhea**	2	0	0	0	0	3	0	0	0	0
**Vomiting**	3	2	0	0	0	3	1	0	0	0
**Constipation**	4	1	0	0	0	5	1	0	0	0
**Cardiac function**	0	0	0	0	0	0	0	0	0	0
**Pneumonia (X-ray)**	2	0	0	0	0	2	0	0	0	0
**Peripheral numbness**	0	0	0	0	0	0	0	0	0	0
**Allergic reaction**	2	0	0	0	0	3	0	0	0	0
**Abdominal ache (postoperation >7 days)**	7	1	0	0	0	8	1	0	0	0

NCI-CTCAE, National Cancer Institute common terminology criteria for adverse events; WBC, white blood cell.

## Discussion

This study evaluated the safety of intraperitoneal chemotherapy with raltitrexed in gastric cancer patients, comparing the chemotherapy with a placebo in the form of normal saline. Results showed that this chemotherapy is a safe method of treating these patients.

Lymphatic metastasis and intraperitoneal dissemination of cancer cells are two main reasons for gastric cancer recurrence after radical resection. Peritoneal implantation metastasis could be caused by the direct spread of the gastric cancer cells or breakage of lymphatic metastasis. With the advance of related technologies, many methods for measuring micrometastatic cancer cells can also be used for the determination of the clinical stages, pathological types, and other features involved in the peritoneum metastasis of gastric cancer, and these have demonstrated that most patients have peritoneal metastasis of gastric cancer [2, 5, 12].

Studies have reported that intraoperative rinse and perioperative intraperitoneal chemotherapy can effectively eliminate the presence of free cancer cells [22] and improve the 5-year survival rate. Peritoneal perfusion during operation has been regarded as an effective method in treating gastric cancer. Studies have demonstrated that intraperitoneal injection could increase the efficacy of medication to ten- or even 1000-fold that of intravenous injection [23] and that blood concentrations of drugs are related to the ability to eliminate cancer cells. A previous study has estimated that chemotherapeutic drugs could eliminate ten times the number of cancer cells when drug concentrations at the tumor/target site are increased at one time point [24].

The peritoneal-plasma barrier can decrease the clearance of chemotherapeutic drugs and thus increase the drug treatment time, which could effectively increase the damage to the cancer cells caused directly by the drugs. In addition, chemotherapeutic drugs could be absorbed by the peritoneum and enter the circulation system through the portal system and the retroperitoneal lymphatic system, which is consistent with the metastatic pathway of gastric cancer, and thus could increase the possibility of eliminating micrometastatic lesions in the lymphatic system and liver, and in turn, reduce the risk of hepatic metastases.

Many drugs including Melphalan, 5-FU, Mitoxantrone, Adriamycin, and Topotecan have been used in intraperitoneal chemotherapy; however, some other drugs including cisplatin, Paclitaxel, and Carboplatin are more commonly used in clinical practices for the treatment of gastric cancers, especially cisplatin, which has the most comprehensive clinical data. Studies have reported that the concentration of cisplatin on the surface of the tumors is ten- to 20-fold greater when administered intraperitoneally versus intravenously, whereas the drug concentration in the peripheral blood is significantly lower than when administered intravenously. The severe adverse effects of cisplatin have also been a challenge for clinicians. Long-term use of cisplatin can induce resistance in most patients, which could increase the recurrence rate of the cancer. The relatively low molecular weight of cisplatin allows rapid absorption of this drug into blood, which could increase the systematic adverse effects. Recently, several studies have focused on investigating embedding cisplatin in liposomes by fibrin sealant or other sustained-release matrix materials to reduce excretion of the drug. Currently, rinsing the abdominal cavity with 5-FU or even distilled water is widely applied as a part of radical resection of gastric cancer. As a first-line chemotherapy drug for gastrointestinal cancer, 5-FU has also been chosen as a chemotherapy drug for intraperitoneal chemotherapy [25, 26]. As a sensitive anti-metabolism drug, 5-FU needs to be metabolized to activate metabolites after absorption and then eliminate the cancer cells [1, 27].

The clearance of drugs is dependent on two factors, namely the characteristics of the drug and peritoneum. Drugs with higher molecular weight generally have lower fat-solubility, and are cleared from abdominal cavity more slowly, which could increase the reaction time between the drug and tumor and allow the drugs to penetrate deep into the tumor tissues. The molecular weight of raltitrexed is 458, which is much higher than that of cisplatin and 5-FU; in addition, the half-life of raltitrexed is 196 hours; the long half-life of this drug allows it to react with cancer cells for a long time without using a slow-release drug matrix. However, no previous study has investigated the safety and efficacy of intraperitoneal chemotherapy with raltitrexed.

Raltitrexed is a specific inhibitor of water soluble thymidylic acid synthase, which can be actively absorbed by cells through cell membrane carriers in its reduced form of methotrexate and can be metabolized into different polyglutamic acids within the cells that react with cancer cells for a long time with higher efficacies than raltitrexed. The long half-life of raltitrexed makes it a promising candidate of intraperitoneal chemotherapeutic drug.

Previously, raltitrexed has been used as a postoperative chemotherapeutic drug for colorectal, gastric, and breast cancers in combination with oxaliplatin instead of 5-FU [28, 29].

This study has some limitations. The relatively small sample size of the present study limited the statistical power of the study; thus these findings should be interpreted with caution, and further studies with larger sample sizes are warranted to validate our findings. This study is just a preliminary study on the safety of the use of raltitrexed; the effectiveness of this method of chemotherapy needs to be fully evaluated in longer term study. In addition, we used the recommended dose of raltitrexed for injection; further studies are needed to investigate the pharmacokinetics and pharmacodynamics of raltitrexed while using it as an intraperitoneal chemotherapeutic drug.

To conclude, we believe that raltitrexed could be better suited for intraperitoneal chemotherapy than 5-FU given its unique characteristics. It showed no obvious local irritation symptoms (including peritoneal inflammatory response, substantially increased drainage volume, or adhesive intestinal obstruction) when 2 mg of raltitrexed was used, or when the dose was increased to 4 mg for further analysis.

## Conclusions

The findings of the present study showed that there were no significant differences in the postoperative gas passage time, mean drainage volume (3 days after the operation), and other complications (including incision infection, pulmonary infection, anastomotic leakage, fever for more than 3 days after the operation, postoperative drainage volume, peritoneal irritation signs, and postoperative diarrhea) between the two groups, suggesting that intraperitoneal chemotherapy with raltitrexed did not increase the risk of the operation. In addition, no significant difference was found while comparing hematologic toxicity, renal toxicity, hepatotoxicity, and cardiac disease parameters before and at 7 day after the operation, in each of the two groups. The toxic and adverse events were all graded as I or II according to **NCI-CTCAE v3.0** and therefore were mild or moderate. Furthermore, no systematic drug reaction was found in the patients who received raltitrexed, which could be because only limited amounts of the drug could be absorbed into the blood or lymphatic system, and thus only a low dose of the drug could reach other organs; therefore, the effects and side-effects of intraperitoneal chemotherapeutic drugs are mainly focused within the abdominal cavity, and induce only limited systematic adverse effects. The findings of the present study demonstrate that intraperitoneal chemotherapy with raltitrexed shows good safety.

## Abbreviations

ALT: alanine aminotransferase
BUN: blood urea nitrogen
LDH: lactate dehydrogenase
NCI-CTCAE: National Cancer Institute common terminology criteria for adverse events
NS: normal saline
PCR: polymerase chain reaction
RBC: red blood cell
WBC: white blood cell.
